# Ellipsometric Identification of Transition from a Layered Metal-Dielectric Film to a Hyperbolic Metamaterial

**DOI:** 10.1021/acsaom.5c00534

**Published:** 2026-01-12

**Authors:** Samhita Kattekola, Vinod M. Menon, Alexander Couzis, Ilona Kretzschmar

**Affiliations:** † Department of Chemical Engineering, 14770 The City College of New York, CUNY, New York, New York 10031, United States; ‡ Department of Physics, 14770 The City College of New York, CUNY, New York, New York 10031, United States

**Keywords:** hyperbolic metamaterial, ellipsometry, characterization, silver-alumina, layered thin films, transfer matrix method, hyperbolic metamaterial design, e-beam evaporation

## Abstract

Hyperbolic metamaterials (HMMs) continue to be intriguing due to their applications in super-resolution imaging and spontaneous emission control. One of the successful realizations of HMMs is a layered metal-dielectric film. Despite the extensive knowledge in thin-film technology and the promises of HMM’s applications, the scale-up and practical utilization of HMMs have not yet occurred. A general design approach is needed to predict the transition of a layered structure into an HMM. In this work, effective medium theory and the transfer matrix method are combined to determine the transition and validated by spectroscopic ellipsometry measurements on a predefined HMM structure made of silver and alumina. Four interdependent design parameters are explored: thicknesses of metal and dielectric layers, transition wavelength, and the minimum number of periods required for a layered metal-dielectric film to display hyperbolic dispersion. The findings are presented as a practical engineering design chart, similar to a state diagram, which can be extended to other combinations of materials.

## Graphical abstract

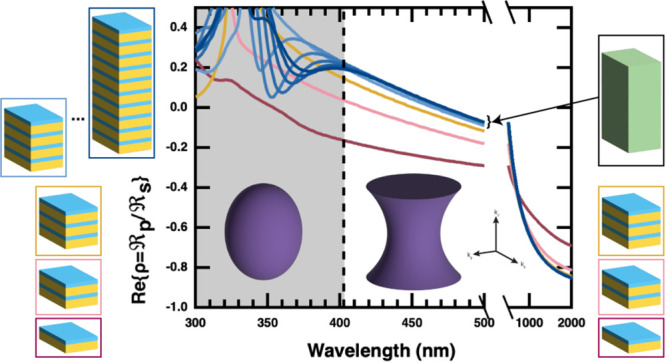

## Introduction

Interactions between metals and dielectrics have been utilized to create interesting optical effects since ancient Roman times.^1^ The history of thin inorganic films is particularly rich, spanning nearly 5000 years of technological innovation.^2,3^ The advent of vacuum technology has further enriched the utilization of thin films.^3,4^ Although practically used for a variety of applications ranging from decoration to mirrors throughout history, the theoretical framework of optics for metal–dielectric systems was established by Paul Drude only in the late 19th century.^5^ Subsequently, the discovery and innovation of modern optical systems evolved exponentially, leading (but not limited) to hyperbolic metamaterials in the early 21st century.^6^

Metamaterials, initially theorized as “left-handed” materials in 1968 by Vesalago,^7^ are non-naturally occurring materials with negative electric permittivity, ε, and negative magnetic permeability, μ. The past two decades have seen an exponential rise in the discovery of metamaterials.^8^ The coining of the term “metamaterials” and evolution of metamaterials, from the earliest realization in the form of split-ring resonators in the 1990s to modern metasurfaces, has been reviewed by Ali et al.^9^ Metamaterials have been fashioned into metadevices^10–13^ and are starting to be explored in the colloidal space.^13–18^

Hyperbolic metamaterials (HMMs) are a class of metamaterials that have garnered interest because of their applications in super-resolution imaging^19^ and spontaneous emission control^20^ among others. HMMs comprise metal and dielectric domains arranged in a specific subwavelength configuration to tailor desired optical properties. The theory, design, and plasmonic character have been reviewed extensively by Poddubny et al.,^21^ Shekhar et al.,^22^ and most recently, by Ramírez-Aragón et al.^23^

HMMs are uniaxial anisotropic materials with a tensorial effective electric permittivity, as shown in eq 1, whose parallel (ε_∥_) and perpendicular (ε_⊥_) components have opposite signs.^6^ This is in contrast to isotropic materials, where ε_∥_ = ε_⊥_, or anisotropic materials, where ε_∥_ and ε_⊥_ are both either positive or negative. The dispersion of light in a medium described by the optical isofrequency curve (IFC) is given in eq 2, where *k*_*x*_, *k*_*y*_, and *k*_*z*_ are the *x*, *y*, and *z* components of the wave vector, *k*, respectively, ω is the angular frequency, and *c* is the speed of light. HMMs display hyperbolic dispersion; i.e., the shape of the resulting IFC for an HMM is an open hyperboloid surface, in contrast to the usually observed closed ellipsoidal surface.

$$\varepsilon_{\mathrm{eff}}=\left[\begin{array}{lll} \varepsilon_{xx} & 0 & 0\\ 0 & \varepsilon_{yy} & 0\\ 0 & 0 & \varepsilon_{zz} \end{array}\right]=\left[\begin{array}{lll} \varepsilon_{∥} & 0 & 0\\ 0 & \varepsilon_{∥} & 0\\ 0 & 0 & \varepsilon_{⊥} \end{array}\right]$$ 1

$$\frac{k_{x}^{2}+k_{y}^{2}}{\varepsilon_{⊥}}+\frac{k_{z}^{2}}{\varepsilon_{∥}}=\frac{\omega^{2}}{c^{2}}$$ 2

As the electric permittivity changes with wavelength, the value of the real part of ε_∥_ and ε_⊥_ determines the regime in which a material has elliptical vs hyperbolic dispersion.^24^ The material has hyperbolic dispersion when either ε_∥_ or ε_⊥_ is negative. When ε_∥_ is positive and ε_⊥_ is negative, the IFC is a one-sheet hyperboloid (Type I HMM), whereas a positive ε_⊥_ with negative ε_∥_ produces a two-sheet hyperboloid IFC (Type II HMM).^22^

Figure 1 shows the real part of the components of electric permittivity as a function of wavelength for a uniaxial anisotropic material, accompanied by graphical representations of the IFC for each regime. The dashed line shows the variation in ε_∥_, whereas the dotted line shows the variation in ε_⊥_ as a function of wavelength. The transition points, indicated by three vertical solid lines, represent the wavelengths at which the IFC transitions first from elliptical dispersion to Type I HMM (λ_ellip–TypeI_ ≈ 325 nm), then back to elliptical dispersion (λ_TypeI–ellip_ ≈ 340 nm), and last from elliptical dispersion to Type II HMM (λ_ellip–TypeII_ ≈ 353), respectively. The transition wavelengths and regimes observed strongly depend on the realization of the HMM. It is possible to have all three transitions as shown in Figure 1, two transitions of either elliptical to Type I to Type II HMM or elliptical to Type II to Type I HMM, or only one transition of either elliptical to Type I or elliptical to Type II.^25^ One of the common realizations for HMMs is a film with alternating metal and dielectric layers, the effective electric permittivity of which depends on the fill fraction, Φ.^22^ Metal-dielectric films that exhibit Type II hyperbolic dispersion (hereafter referred to as HMM character) are the focus of this research. Figure 1 is such an example, where Φ = 0.5. The wavelength at which the transition to Type II occurs is labeled as λ_HMM_ (thick vertical solid line in Figure 1).

**Figure 1:**
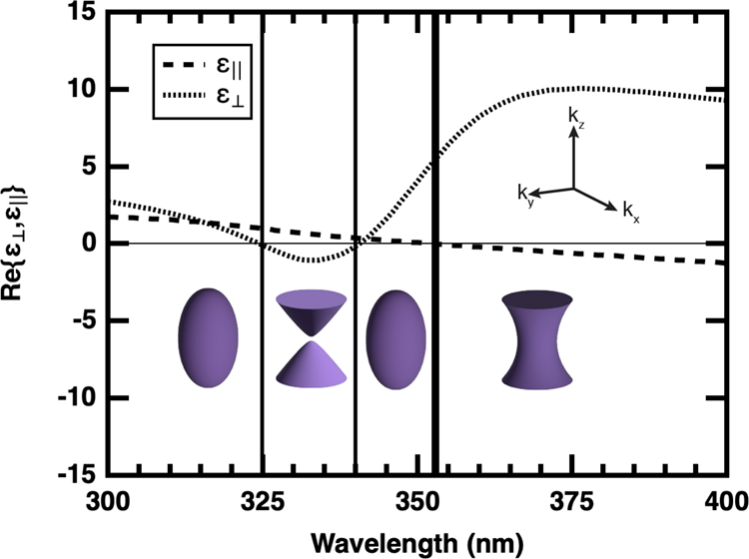
Evolution of the real part of the electric permittivity components from a material with elliptical dispersion to a hyperbolic metamaterial generated using optical constants obtained in this work for a metal thickness of 10 nm and dielectric thickness of 10 nm (Φ = 0.5). ε_⊥_ and ε_∥_ are represented by dotted and dashed lines, respectively. The three vertical solid lines indicate the wavelengths at which the material first transitions from elliptical dispersion to Type I HMM, back to elliptical dispersion, and then to Type II HMM (thicker vertical solid line, λ_HMM_), respectively. The insets show graphical representations of the iso-frequency contour (IFC) for the respective regimes and the wave vector reference axis orientation.

Abundant literature exists on growing layered thin films, which emphasizes the interest in these novel materials.^4^ Research over the past decade has focused on developing novel Type II HMMs, characterizing their optical response, identifying potential applications, and generally pushing the limits of HMMs’ usage. The first experimental demonstration of a Type II HMM was reported by Liu et al.^26^ in the form of a hyperlens that surpassed the diffraction limit of light using a curved surface made of silver and alumina layers. Work by Hoffman et al.^27^ showed negative refraction by layering doped semiconductor films. Krishnamoorthy et al.^24^ introduced topological transitions using silver and titanium oxide layers, showing an increase in the spontaneous emission rate of quantum emitters in such structures. For additional combinations, designs, and applications, the reader is referred to the reviews by Poddubny et al.^21^ and Cortes et al.^25^

The common design parameters in the literature for the fabrication of HMMs with alternating metal-dielectric layers are metal thickness, *h*_*m*_, dielectric thickness, *h*_*d*_, the electric permittivity of the metal and dielectric material, ε_*m*_ and ε_*d*_, respectively, the fill fraction of metal in the system, Φ (eq 3), and the transition wavelength for the system to become an HMM, λ_HMM_. The design parameters are generally chosen based on effective medium theory (EMT) calculations.^28^ Effective medium implies that the film made of metal-dielectric layers behaves as a homogeneous material with an effective electric permittivity; ε_∥_ and ε_⊥_ are then calculated using eqs 4 and 5, respectively.

$$\Phi =\frac{h_{m}}{h_{m}+h_{d}}$$ 3

$$\varepsilon_{∥}=\Phi \varepsilon_{m}+\left(1-\Phi \right)\varepsilon_{d}$$ 4

$$\varepsilon_{⊥}=\frac{\varepsilon_{m}\varepsilon_{d}}{\Phi \varepsilon_{d}+\left(1-\Phi \right)\varepsilon_{m}}$$ 5

Essentially, for a given combination of metal and dielectric, λ_HMM_ is a function of Φ (Figure 1). However, λ_HMM_ and Φ are insufficient in identifying the number of periods at which the material transitions from a metal-dielectric layered structure into an HMM. For instance, consider Figure 1, which has a Φ = 0.5, predicts the same solution (λ_HMM_ ≈ 353 nm) for a 5:5 and a 10:10 nm metal:dielectric layer ratio. However, the 5:5 structure will likely need a larger number of periods to become an HMM. EMT simply does not take into account the impact of the number of periods in the film or total film thickness, both of which impact whether the resulting film is an HMM.^25,29^ This represents a limitation of EMT’s applicability in the predictive design of HMMs and increases the reliance on complex optical setups to identify if the material produced exhibits HMM character only after it is fabricated.^21^

A typical workflow found in the literature involves choosing the size and combination of materials (*h*_*m*_ and *h*_*d*_) most suited for the desired application. Consequently, λ_HMM_ is obtained by the utilization of optical constants available in the literature, EMT calculations (eqs 3–5), and construction of the corresponding Figure 1.^24^ Then, the constituent metal and dielectric films are grown, characterized to obtain actual thicknesses and optical constants using ellipsometry, and λ_HMM_ is recalculated for the actual materials made. Next, samples of at least four periods (but also as many as 8 or 200 periods have been reported)^26,29–31^ are grown, and it is not apparent from the literature how the number of periods is chosen. Subsequently, optically active materials such as quantum dots are coupled to the material to investigate its optical properties.^22,32,33^ It is only in the last step, which requires complicated optical and photonics setups, that the material is confirmed to be an HMM, posing practical limitations on the utilization of HMMs. The review by Lee et al.^34^ summarizes complex experiments and calculations that have been performed for HMM realizations and concludes with suggestions to extend the range of practical applications of HMMs.

The complexity in HMM characterization methods mentioned above has led to a search for simpler approaches such as reflectometry and ellipsometry to identify HMM character.^35–40^ Ellipsometry,^41,42^ one of the most commonly used techniques for characterization of inorganic and organic thin films, has only been sparingly used for HMM characterization. The complexity in modeling HMM using ellipsometry primarily arises from the difficulty in modeling the out-of-plane electric permittivity component. Several works have focused on estimating the out-of-plane permittivity and fitting them to the experimental data as uniaxial models.^30,35–38,43^ Prominent works that experimentally determine both in- and out-of-plane permittivity are those by Tumkur et al.,^35^ Kelly et al.,^30^ and Zhang et al.^43^ They assume that the materials being characterized are uniaxial homogeneous materials and compare them to theoretical calculations to discern the effective permittivity of the HMM. Tumkur et al.^35^ and Kelly et al.^30^ utilize anisotropic and general spectroscopic ellipsometry measurements, respectively, whereas Zhang et al.^43^ employ isotropic spectroscopic ellipsometry measurements to obtain the effective permittivity. A key distinction in Zhang et al.^43^ is the measurement of the sample itself as they use total internal reflection ellipsometry, requiring an index-matched prism^44^ to increase the sensitivity of the out-of-plane interactions to robustly extract the out-of-plane permittivity coefficients.

Here, we posit the following question: Can ellipsometry, a fundamentally far-field method, be used to distinguish an HMM from a multilayer film? If so, can it be used to predict the transition of a layered metal-dielectric film into a hyperbolic metamaterial? To answer these questions, a previously verified and known hyperbolic metamaterial^32^ comprising silver and alumina layers with seven periods (**7P**) is fabricated. Additionally, one period (**1P**) and four period analogues (**4P**) are also grown. All films are characterized and analyzed using isotropic spectroscopic ellipsometry measurements. AFM is used to independently confirm film thicknesses. Subsequently, ellipsometry data are compared to numerical simulation predictions based on a 2 × 2 transfer matrix method (TMM). Next, the simulations are used to predict the ellipsometric response for the Ag–Ge/Al_2_O_3_ system as a function of the number of periods, revealing the threshold for HMM behavior. A state diagram is constructed that allows prediction of the HMM transition using four interdependent design parameters: metal layer thickness, *h*_*m*_, dielectric layer thickness, *h*_*d*_, minimum number of periods of metal-dielectric layers in the film, *P*_HMM,min_, and the wavelength at which the transition into HMM occurs, λ_HMM_. The resulting design chart enables the tailoring of hyperbolic metamaterials for specific applications such as hyperlens and enhanced spontaneous emission.

## Experimental and Simulation Details

### Material Fabrication

Samples are fabricated using an e-beam evaporator (AJA International Orion 8E Evaporator) maintained at *P* < 10^–8^ Torr and equipped with a quartz crystal microbalance to monitor thickness (reported as nominal thickness). All films are grown at an average rate of 0.3 Å/s. Alumina (Al_2_O_3_), silver (Ag), and germanium (Ge) targets used are purchased from Kurt J Lesker. Single-side polished (SSP) silicon wafers purchased from University Wafer, Inc. are used as substrates. Substrates are cleaned by submerging them in a mixture of Nochromix and sulfuric acid for 2 h, followed by copious rinsing with deionized water and drying in an oven at 70 °C. Any sample containing silver requires deposition of a germanium seed layer (nominal thickness of 2 nm) to allow layered film growth.^45^ Additionally, whenever the last layer of the sample is silver, a protection layer of alumina (nominal thickness of 6 nm) is grown to prevent silver oxidation after removal from the evaporator.

Figure 2 shows schematics of the layered films fabricated on silicon wafer substrates. Three samples of each film are grown. The cross-section of the film is shown in Figure 2A. One period of the film has a nominal thickness of 27 nm, comprising 15 nm alumina, 2 nm seed layer of germanium, and 10 nm of silver. Since the period ends in silver, a 6 nm protection layer of alumina is deposited on all layered films. Figure 2B shows the three-layered metal-dielectric films comprising (i) one (**1P**), (ii) four (**4P**), and (iii) seven (**7P**) periods. Table 1 summarizes the nominal thicknesses for all samples. In addition, alumina films with five nominal thicknesses (3, 6, 10, 15, and 20 nm) and germanium–silver-alumina films with four silver nominal thicknesses (5, 10, 15, and 20 nm) are grown for ellipsometry calibration.

**Figure 2:**
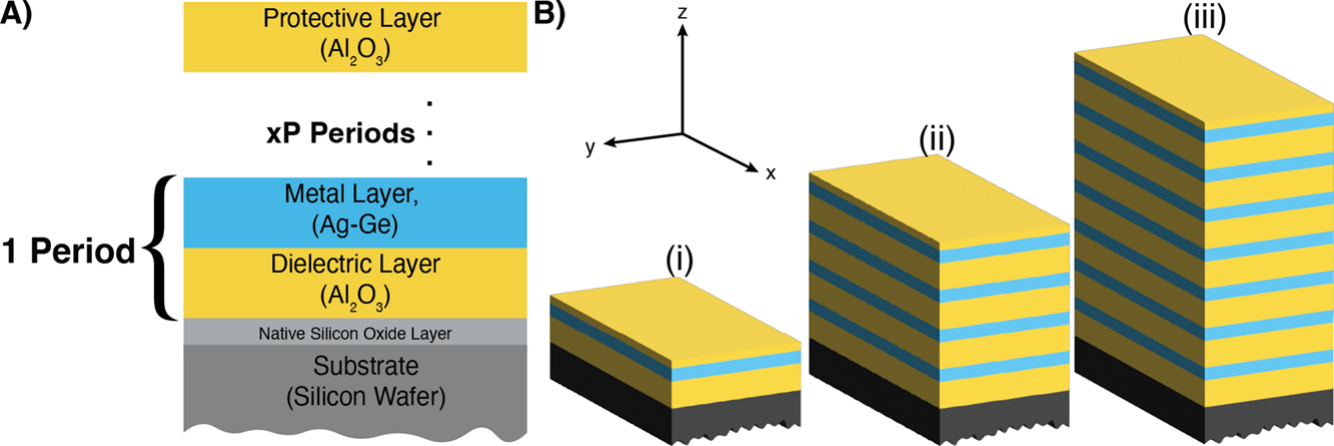
Schematics of layered films. (A) Cross-section of fabricated periodic layered metal-dielectric film. One period consists of a dielectric layer (DL, yellow) of alumina (Al_2_O_3_) and a metal layer (ML, blue) of silver grown on a seed layer of germanium (Ag–Ge). All films are capped with a protection layer (PL, yellow) of Al_2_O_3_. A silicon wafer with a native oxide layer is used as the substrate (gray and light gray, respectively). (B) Schematic of (i) **1P**-, (ii) **4P**-, and (iii) **7P** films.

**Table 1:** Sample Nominal Thicknesses Read from an E-Beam Evaporator Thickness Monitor (in nm)

sample	*N* ^a^	total alumina^b^	total germanium	total silver	total^b^
**1P**	1	21	2	10	33
**4P**	4	66	8	40	114
**7P**	7	111	14	70	195

a *N* is the number of Ag–Ge/Al_2_O_3_ periods.

b Value includes a 6 nm Al_2_O_3_ protection layer.

### Ellipsometry Measurements

A J.A. Woollam Co, Inc. V-VASE Ellipsometer equipped with WVase Software^46^ is used to conduct variable angle spectroscopic ellipsometry (SE). Isotropic ellipsometry measurements for all samples are done over a wavelength range of 300–2000 nm with a step size of 30 nm (10 nm step for silicon wafer substrate) for two incident angles (65° and 75°). The ellipsometer measures the ratio of Fresnel reflection coefficients, $R_{p}$ and $R_{s}$ , for *p*- and *s*-polarization, respectively. The measurement is reported as either Ψ and Δ or ρ, which are interrelated as given in eq 6.

$$\rho =\frac{R_{p}}{R_{s}}=\tan\;\Psi e^{i\Delta }$$ 6

The J.A. Woollam Co., Inc. CompleteEASE Software^47^ is used for modeling of optical constants and for regression analysis to obtain layer thicknesses and a goodness-of-fit, which is reported as root-mean-square error (MSE).^47^ The description of MSE and its relation to Ψ and Δ are shown in SI Section I.2.1.

The modeling of the films is done in four consecutive stages. The cleaned silicon wafer substrate is modeled first using the native oxide model provided by the Woollam CompleteEASE Software (MSE and native oxide thickness reported in SI Section I.2.2). This model is used as a substrate layer without adjustment for all further modeling. Next, the alumina layer is modeled by modifying the CompleteEASE built-in Cauchy model for Al_2_O_3_ to reflect the optical properties of alumina deposited using e-beam evaporation.^48,49^ The silver layer is defined as a composite Ag–Ge layer and modeled using the CompleteEASE general oscillator model. A combination of Drude, Lorentz, and Cody-Lorentz oscillators is used to reflect the layer of silver with the germanium seed layer.^50^ The reported optical constants for individual layers of Al_2_O_3_ (Figure S4) and Ag–Ge (Figure S6) are checked to be consistent with those reported in the literature. MSE and thickness calibration details for the individual layers are provided in SI Sections I.2.3 and I.2.4, respectively. Based on the optical constants and layer thicknesses, the parallel and perpendicular components of the electric permittivity tensor for Al_2_O_3_ and Ag–Ge system studied here are predicted using eq 3 (SI Section I.3, Figure S7). Subsequently, the layered films are modeled as a multilayer model with a silicon wafer substrate and alternating Al_2_O_3_ and Ag–Ge layers culminating in the Al_2_O_3_ protection layer (see Figure 2). Here, a single period comprises two layers, Al_2_O_3_ and Ag–Ge. For films with more than one period, a technique known as parameter coupling is employed to keep the number of fitting parameters in the model constant.^51^ Parameter coupling is a built-in feature of CompleteEASE, wherein the relationship between two layer parameters is defined. It assumes that every alternative repeating layer has the same thickness and optical constants. This assumption truly represents the periodic multilayer character of the film as fabricated. The reduction in fitting parameters simplifies the system and also reduces the error propagation from multiple fit parameters. The data for three independently fabricated samples of each film are simultaneously fitted for both angles of incidence and reported in Table 2.

**Table 2:** Fitted Layer Thicknesses, *h*_*x*_, from Ellipsometry for the Dielectric Layer (*x* = DL, Al_2_O_3_), Metallic Layer (*x* = ML, Ag–Ge), Protection Layer (*x* = ML, Al_2_O_3_), and Total Film Thickness (*x* = total, SE) and Measured Total Thickness from AFM, *h*_total,AFM_ ^a^

sample	MSE	*h*_DL,Al_2_O_3__	*h*_ML,Ag–Ge_	*h*_PL,Al_2_O_3__	*h*_total,SE_	*h*_total,AFM_
**1P**	10.0	21.1 ± 0.3	10.84 ± 0.03	8.82 ± 0.09	40.8 ± 0.3	39.0 ± 0.8
**4P**	15.8	23.7 ± 0.1	11.17 ± 0.05	8.2 ± 0.1	147.6 ± 0.2	128 ± 1
**7P** ^b^	23.2	25.6 ± 0.2	14.0 ± 0.1	9.8 ± 0.2	284.0 ± 0.7	209 ± 1

a All thickness values in nm reported with one standard deviation; MSE is the root mean square error reported by CompleteEASE.

b Note that the thickness values obtained from the **1P** model (row 1) are used as the initial guess for the **7P** data fit (see text).

### AFM Measurements

Atomic force microscopy (AFM) is used on selected calibration and film samples to confirm thickness measurements predicted by ellipsometry. A Bruker Dimension FastScan atomic force microscope is used in contact mode with a ContAI-G AFM Probe from BudgetSensors. To enhance accuracy of the AFM thickness measurement, a subset of films is deposited on a 4 μm silica particle submonolayer that is subsequently lifted off. The silica particles shadow the substrate from the depositing material and leave behind an empty area on the substrate. The boundary between the shadowed area and the deposited film allows accurate measurement of the film thickness (see SI Section I.1.2). AFM measurements are reported along with ellipsometry calibration data in SI Section I as well as with **1P**, **4P**, and **7P** film data in Table 2.

### Numerical Simulations

MATLAB^52^ is used to simulate the real (Re­{ρ}) and imaginary (Im­{ρ}) parts of the ellipsometric parameter, ρ, for combinations of Ag–Ge and Al_2_O_3_ layer thicknesses ranging from 1 to 20 nm and 1 to 40 nm, respectively, with a resolution of 0.01 nm. The wavelength range for the simulation is 300–2000 nm with a step size of 1 nm. In order to discern the HMM character, Re­{ρ} is calculated for all combinations at λ_HMM_ for films with 1 to 150 periods. Re­{ρ} values from films with 100–150 periods are averaged and reported as Re­{ρ_*∞*_}. The average difference between Re­{ρ_*∞*_} and the highest Re­{ρ} value for the entire parameter space investigated is reported as the confidence interval δRe­{ρ} = ± 0.035. The TMM with 2 × 2 Abeles’ formulation as outlined by Born and Wolf^53^ is utilized. The optical constants for Ag–Ge and Al_2_O_3_ obtained from ellipsometry measurements are used in the simulations. Details of the calculations are given in SI Section II.

## Results and Discussion

Three types of films with one (**1P**), four (**4P**), and seven (**7P**) periods on silicon wafer substrates are investigated. A multilayer model is constructed to characterize and determine the thickness of individual layers constituting the **1P** film. The accuracy of the multilayer model is validated with AFM measurements. Then, the model is extended to the **4P** and **7P** films using parameter coupling to assess the model’s ability to correctly represent the films with an increased number of periods. Numerical simulations are performed to validate the experimental findings and discern the emergence of the HMM character. A universal HMM design chart is built for the Al_2_O_3_/Ag–Ge system that reveals the minimum number of periods, *P*_HMM,min_, and transition wavelength, λ_HMM_, needed for an Al_2_O_3_/Ag–Ge system to exhibit HMM character.

### Ellipsometry Characterization of Layered Films

**1P**, **4P**, and **7P** samples grown on silicon wafers (Figure 2) are characterized using spectroscopic ellipsometry (SE) employing multilayer modeling and parameter coupling together with the optical constants obtained from modeling Al_2_O_3_ and Ag–Ge films (described in experimental details and in SI Section I).

#### **1P** Film Modeling

Figure 3 shows the ellipsometric parameters, Ψ and Δ, as blue and red circle markers, respectively, and their corresponding multilayer model fits (solid lines) for the **1P** film on a silicon wafer as a function of the wavelength, λ. The shaded blue and red regions (smaller than marker size) represent one standard deviation for the average of three samples. The filled and open markers correspond to incident angles of 65° and 75°, respectively. The multilayer model is comprised of three layers, i.e., an alumina layer (DL), a silver layer (ML), and an additional alumina protective layer (PL), as shown in Figure 2Bi. The three nominal thicknesses (given in Table 1) are used as initial guesses for the iterative modeling.

**Figure 3:**
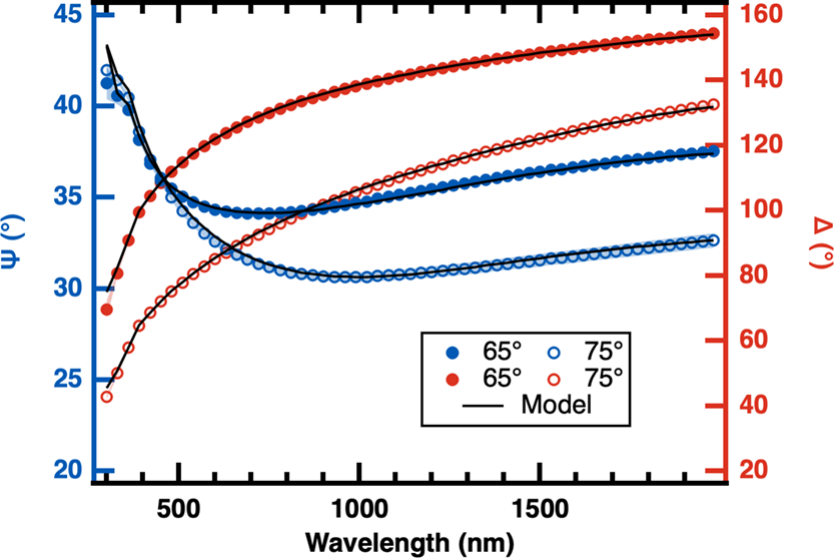
Ellipsometric parameters Ψ (blue, left axis) and Δ (red, right axis) as a function of wavelength for the **1P** film obtained at 65° (filled circles) and 75° (open circles) incident angles. One standard deviation obtained from averaging three independently grown samples is represented as shaded area but is smaller than the marker size throughout. Solid lines represent the best multilayer model fit obtained using CompleteEASE Software (see text).

The first row of Table 2 shows the thickness values obtained from **1P** film fitting. The low overall MSE of 10.0 validates the use and accuracy of the multilayer model for the **1P** film. The total thickness of 40.8 ± 0.3 nm predicted by the multilayer model of the **1P** film (Column 6 in Table 2) agrees with the AFM measured thickness of 39.0 ± 0.8 nm (Column 7 in Table 2) and independently validates the **1P** model and optical constants, allowing calculation of the Figure 1 equivalent for the system studied here (SI Section I.3, Figure S7). Next, the validated **1P** multilayer model is used to fit the ellipsometry data obtained from the **4P** and **7P** films.

#### **4P** and **7P** Film Modeling

Figure 4 shows the ellipsometric parameters, Ψ and Δ, their corresponding standard deviation, and a multilayer model with parameter coupling fits for the **4P** (Figure 4A) and **7P** (Figure 4B) samples using the same color and line assignment as used in Figure 3. Diamond markers are used for **4P** and square markers are used for **7P**.

**Figure 4:**
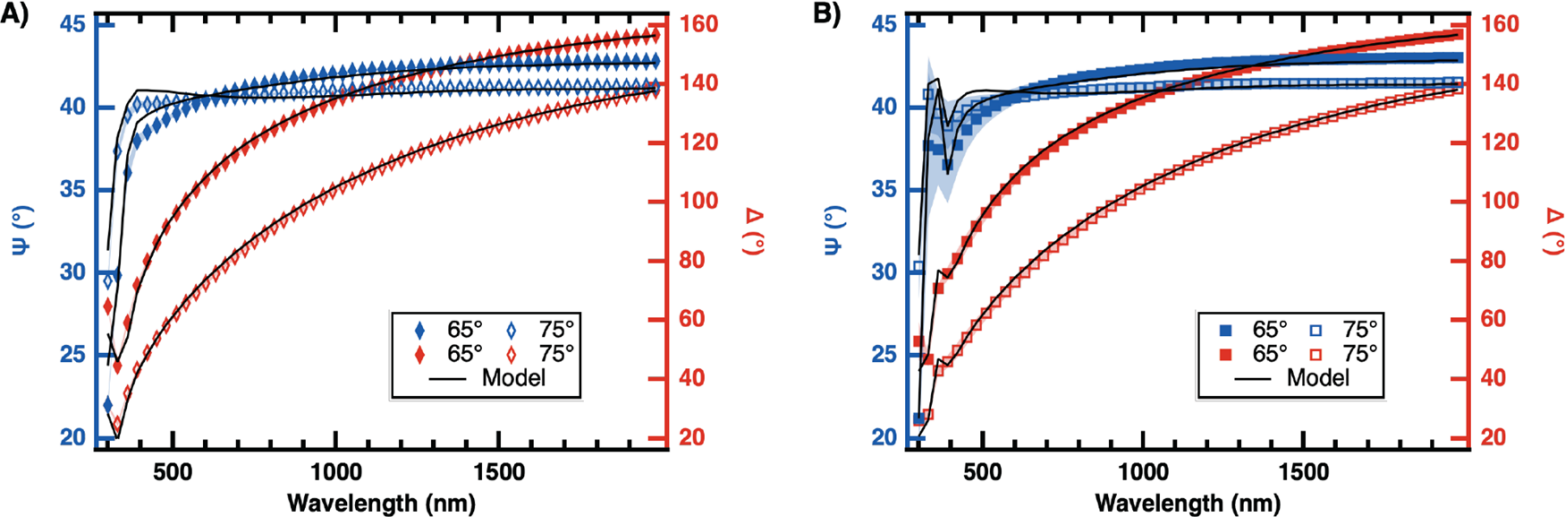
Ellipsometric parameters Ψ (blue, left axis) and Δ (red, right axis) for (A) **4P** (diamond markers) and (B) **7P** (square markers) films as a function of wavelength obtained at 65° (filled markers) and 75° (open markers) incident angles. One standard deviation obtained from averaging three independently grown samples is represented as shaded area and is in some instances smaller than the marker size. Solid lines represent the best multilayer model fit with parameter coupling obtained using CompleteEASE Software (see text).

Comparison of Figures 3 and 4A reveals that the shapes of the Ψ and Δ curves change substantially from the **1P** to the **4P** film. Specifically, the Ψ curve (blue markers) appears flipped, i.e., Ψ decreases with λ in the **1P** film data, whereas it increases with λ in the **4P** film data. Furthermore, there is an apparent shift of the Δ *c*urve to the right, and the range for Δ is slightly increased in the **4P** film data, starting at 20 rather than at 40. In contrast, the Ψ and Δ curves for the **4P** and **7P** films (Figure 4A,B) appear almost identical, with the exception of a peak at λ ≈ 340 nm in the **7P** film data. The apparent changes in both Ψ and Δ between the **1P** and **4P** films indicate that the interaction of light with the films changes as periods are added to the film, while the similarity of the **4P** and **7P** film responses signifies similar optical properties possibly hinting at the emergence of the HMM metamaterial character in as early as the **4P** sample as the **7P** sample has been shown to an be HMM.^32^ The **1P**–**4P** and **4P**–**7P** film differences in Ψ and Δ are shown in SI Section III.

Applying the multilayer model to **4P** and **7P** films requires the addition of either three or six Al_2_O_3_/Ag–Ge periods, respectively, to the **1P** model. The new layers are coupled to each other using the parameter coupling technique (see experimental details) that assigns fitting parameters to each material and fits every layer of the materials as it occurs in the period with the same thickness parameter. Nominal thicknesses (Table 1) are used as initial parameters for fitting the **4P** and **7P** film data. The thickness values predicted by the **4P** and **7P** parameter coupling models are shown in the second and third rows of Table 2, respectively. Note, while the **4P** fit converges with nominal thicknesses as initial parameters, the **7P** fit provides unreasonable fitting parameters (i.e., *h*_PL_ = 5.9 ± 0.2, *h*_ML_ = 7.5 ± 0.1, and *h*_DL_ = 13.9 ± 0.2 nm, MSE = 29.5), resulting in a total thickness of *h*_total_ = 155.7 ± 0.5 nm, which is much smaller (25%) than the **7P** film thickness measured with AFM (*h*_AFM_ = 209 ± 1) nm. A second round of fitting of the **4P** and **7P** film data using the **1P** fitting parameters from Table 2 as initial parameters results in the same fitting parameters for **4P**, but provides a fit with optimized parameters (third row of Table 2) that shows consistent trends with the **1P** and **4P** films and also with the AFM measurement.

Analysis of Table 2 reveals that both *h*_ML_ and *h*_DL_ are overpredicted by the model for the **4P** and **7P** films, while *h*_PL_ is modeled as thinner in the **4P** film and thicker in the **7P** film compared to the **1P** film. Unsurprisingly, comparison of total film thicknesses for the **4P** and **7P** films from ellipsometry fitting (Column 6 in Table 2) with the actual thicknesses measured using AFM (Column 7 in Table 2) also shows a 15 and 36% overprediction for the **4P** and **7P** films, respectively. Interestingly, the MSE values of 15.8 and 23.2, respectively, for the multilayer model with parameter coupling obtained for the **4P** and **7P** films are only slightly higher than the MSE for the **1P** films (10.0), indicating that the model still accurately represents the system.

Generally speaking, in an isotropic material, an increase in material thickness coincides with an increase in the reflectivity of the material. Analogously, a more reflective film will be found to be thicker when fitted. The fact that both *h*_ML_ and *h*_DL_ are overpredicted for the **4P** and **7P** films (Table 2) indicates that the films become more reflective as more periods are added than the total number of periods alone warrants. Transmission data (not shown) for all three films grown on glass substrates show the expected decrease in transmission as the number of periods in the films increases. A key property of Type II HMMs is an increase in the overall reflectivity properties of the material due to their ability to reflect propagating waves and transmit evanescent waves.^25,54^ Thus, the observed overprediction could be a sign of the emergence of a Type II HMM character in the **4P** film. In order to shed light on this behavior, numerical simulations are performed to track the change in ρ (eq 6) as a function of the number of periods.

### Numerical Simulations with TMM

A numerical simulation using the Abeles’ TMM is employed to predict the ellipsometric parameter, ρ, for the **1P**, **4P,** and **7P** films at an incident angle of 65° using their respective fitting parameters from Table 2. Re­{ρ} and Im­{ρ} from the numerical simulations show good agreement with the experimental data (see SI Section IV) and allow prediction of ρ for films as a function of periods.

Figure 5 shows the numerically simulated Re­{ρ} for a system with *h*_PL,Al_2_O_3__ = 8.82, *h*_ML,Ag–Ge_ = 10.84, and *h*_DL,Al_2_O_3__ = 21.1 nm (the **1P** fitting parameters from Table 2), which has a λ_HMM_ ≈ 403 nm using the experimentally obtained optical constants (SI Section I.3, Figure S7). The inset of Figure 5 shows the zoomed-in part of Re­{ρ} from 402 to 406 nm, with λ_HMM_ ≈ 403 nm indicated by the dashed line. As mentioned earlier, the primary characteristic of a Type II HMM is increased reflectivity, shown here as the change in the real part of the ellipsometric parameter ρ. At the transition wavelength (dashed line in Figure 5) beyond which the multilayered structure becomes an HMM, Re­{ρ} continues to increase with the number of periods until it converges into a band. Within the band, Re­{ρ} initially increases further, then decreases until stabilizing at a value toward the lower side of the band, Re­{ρ_*∞*_} (see simulation details). The width of the band is within the confidence interval of 0.035. The fact that the predicted Re­{ρ} for a **4P** film sits at the threshold of this band agrees with the observation that the ellipsometric response from the **4P** and **7P** films is nearly identical and is interpreted as the emergence of the HMM character, i.e., *P*_HMM,min_ = 4 for a *h*_ML,Ag–Ge_/*h*_DL,Al_2_O_3__ combination of 10.84/21.1 nm (Φ = 0.34) with a 8.82 nm Al_2_O_3_ protection layer.

**Figure 5:**
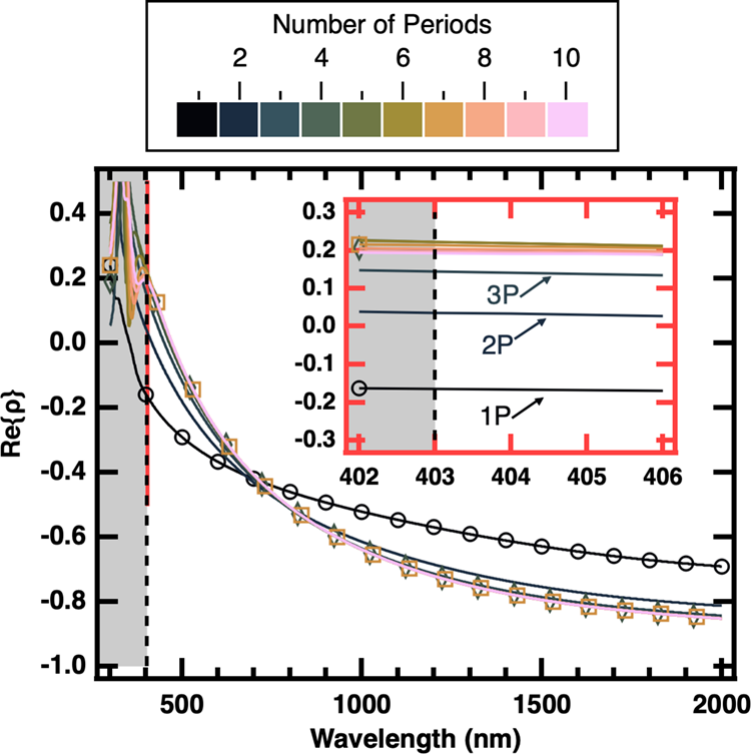
Real part of ρ, Re­{ρ}, as a function of wavelength calculated using the transfer matrix method. Color indicates the number of periods in the film. The dashed line indicates λ_HMM_ ≈ 403 nm, above which the material becomes a Type II HMM. Inset: Zoomed-in image of the region at λ_HMM_ showing the convergence of Re­{ρ} starting with the **4P** film. Open circles, diamonds, and square markers are added to the **1P**, **4P**, and **7P** film Re­{ρ} predictions, respectively.

### HMM Design Chart

Using the observed convergence of Re­{ρ} and the confidence interval (δ*Re*{ρ} = 0.035), the numerical analysis is extended into an HMM design chart for other *h*_ML,Ag–Ge_/*h*_DL,Al_2_O_3__ combinations with a constant Al_2_O_3_ protection layer (*h*_PL,Al_2_O_3__ = 8.82 nm) and an incident angle of 65°, as shown in Figure 6. The maximum thicknesses for the Al_2_O_3_ and Ag–Ge layers in Figure 6 are limited to 40 and 20 nm, respectively, as the experimentally obtained thin-film optical constants may not represent the optical constants of the bulk material.^57,58^ Figure 6 is obtained by first calculating λ_HMM_ for every combination of metal-dielectric thickness using EMT (eqs 3–5). Next, the ellipsometry parameter ρ is simulated for the corresponding λ_HMM_. Last, the period at which δRe­{ρ} first falls within 0.035 of Re­{ρ_*∞*_} (see Figure 5) is identified and denoted as *P*_HMM,min_. Then, λ_HMM_ (colored solid lines) and *P*_HMM,min_ (plateaus in changing gray tones separated by black lines) contour lines are plotted as a function of *h*_DL,Al_2_O_3__ and *h*_ML,Ag–Ge_. The zoomed-in versions of Figure 6 (Figures S12 and S13) revealing the details at low metal or dielectric layer thicknesses and additional versions illustrating various aspects of the design chart (Figures S14 and S15) are provided in SI Section V.

**Figure 6:**
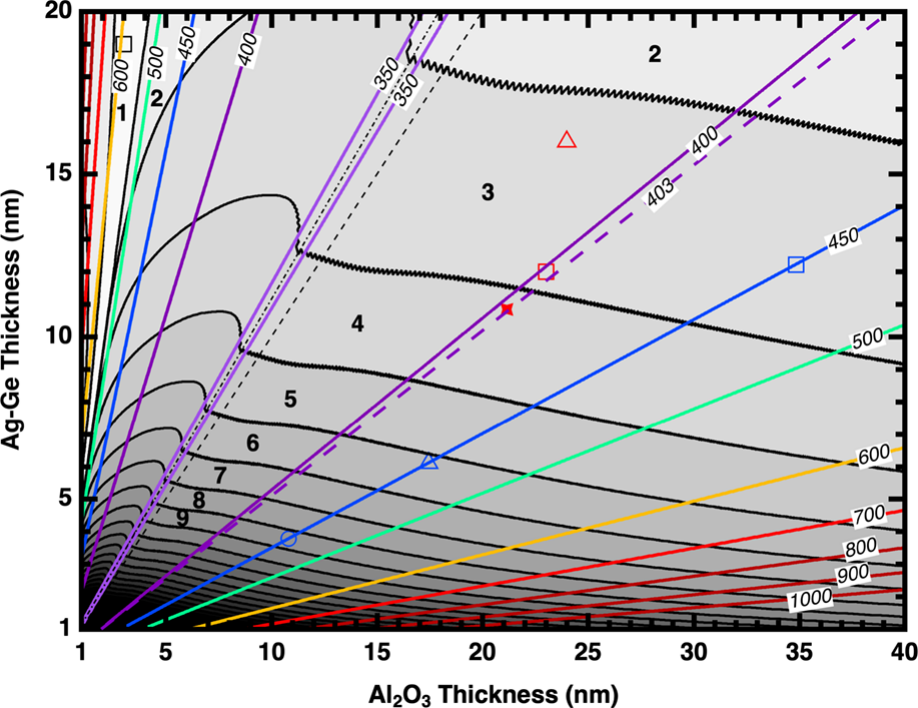
HMM design chart displaying the minimum number of periods, *P*_HMM.min_, and the transition wavelength, λ_HMM_, needed for a multilayer film comprising alternating layers of *h*_DL,Al_2_O_3__ and *h*_ML,Ag–Ge_ to show Re­{ρ} within 3.5% of Re­{ρ_*∞*_} based on numerical modeling. Black solid lines indicate the change in *P*_HMM,min_ by 1 and colored solid lines show selected λ_HMM_ values beyond which the film displays hyperbolic dispersion. The black dashed line indicates the 1:1 ratio of the Al_2_O_3_/Ag–Ge system, whereas the black dot-dashed line indicates the corrected Al_2_O_3_/Ag ratio (see text). The red solid star shows 21.1:10.84 nm Al_2_O_3_:Ag–Ge composition (this work) with a corresponding λ_HMM_ ≈ 403 (colored dashed line). The black open square shows a metal-rich point which needs only one period to be an HMM (see text). Red open triangle^55^ and square^56^ show material compositions discussed in the text from the published literature that show HMM character for three and six periods, respectively. Blue open markers are material combinations shown in Table 3 with λ_HMM_ ≈ 450 nm used to explain the utility of the chart (see text).

As can be seen in Figure 6, λ_HMM_ decreases from the top left corner of the diagram (Ag–Ge-rich films), reaches a minimum at λ_HMM,min_ ≈ 348 nm (dot-dashed black line), and then increases toward the bottom right corner (Al_2_O_3_-rich films). λ_HMM,min_ coincides with the region in the diagram where the Ag–Ge and Al_2_O_3_ layer thicknesses are balanced, i.e., Φ ≈ 0.5. This symmetry of λ_HMM_ as a function of Φ has been discussed in detail by Cortes et al.^25^ They also show in their work that Ag-rich films will transition from an effective metal to a Type II HMM, while Al_2_O_3_-rich films will make this transition from an effective dielectric. In the system discussed here, λ_HMM,min_ occurs at Φ = 0.53 rather than the expected Φ = 0.5 (dashed black line), which is attributed to the presence of the Ge seed layer (2 nm nominal thickness) in the system that supports the growth of smooth silver layers.

*P*_HMM,min_, which is an integer, increases from the top right corner to the bottom left corner of Figure 6, and the solid black lines mark the boundaries at which *P*_HMM,min_ changes by one. A few general trends are observed: (i) as *h*_ML,Ag–Ge_ increases at constant *h*_DL,Al_2_O_3__, *P*_HMM,min_ decreases; (ii) as *h*_DL,Al_2_O_3__ increases at constant *h*_ML,Ag–Ge_, *P*_HMM,min_ also decreases; and (iii) *P*_HMM,min_ is more strongly impacted by changes in *h*_ML,Ag–Ge_. Interestingly, *P*_HMM,min_ changes monotonically with *h*_ML,Ag–Ge_ for all *h*_DL,Al_2_O_3__, but not so when *h*_DL,Al_2_O_3__ is varied at constant *h*_ML,Ag–Ge_. The implication of this feature is that one can make metal-rich and dielectric-rich HHMs with the same *h*_ML,Ag–Ge_ and *P*_HMM.min_ by varying *h*_DL,Al__2__O__3_, allowing studying the impact of *h*_DL,Al_2_O_3__ on HMM properties. Another interesting feature in the metal-rich area of Figure 6 is the narrow sliver (very light gray) of material combinations that require only one period (*P*_HMM,min_ = 1) to start displaying HMM character, e.g., *h*_ML,Ag–Ge_ = 19 nm and *h*_DL,Al_2_O_3__ = 4 nm (black open square marker, see Figure S13). A *P*_HMM,min_ = 1 is plausible because the presence of the protective Al_2_O_3_ layer creates the second metal/dielectric interface needed for the interaction of two evanescent waves in the film.^29^

The utility of Figure 6 is demonstrated by the red and blue markers. The red solid star shows the Ag–Ge combination used in the **1P**, **4P**, and **7P** films studied here, i.e., *h*_DL,Al_2_O_3__ = 21.1 and *h*_ML,Ag–Ge_ = 10.84 nm. The point falls on the *P*_HMM,min_ = 4 plateau and between the λ_HMM_ ≈ 400 nm and λ_HMM_ ≈ 450 nm lines, in good agreement with the similarity in the observed ellipsometry response from the **4P** and **7P** films (Figure 4) and the calculated λ_HMM_ ≈ 403 nm (colored dashed line and Figure S7). The red open triangle shows work by Indukuri et al.,^55^ in which a three-period Al_2_O_3_/Ag antenna (24/16 nm) resulted in a 30-fold enhancement of the overall photoluminescence from a WS_2_ monolayer sample, agreeing with the *P*_HMM,min_ = 3 predicted by Figure 6. The red open square shows the material combination used for a six-period Al_2_O_3_/Ag film (23/12 nm) showing spontaneous emission enhancement for a Coumarin dye, which is further enhanced by an additional silver grating.^56^ This example also agrees with the HMM design chart in that it has six periods, which is larger than the *P*_HMM,min_ = 3 predicted by Figure 6. Note that both of these works do not use the Ge seed layer, which slightly changes the silver optical properties with respect to the reported silver optical constants. Figure 6 can still be utilized in an Al_2_O_3_/Ag system without the Ge seed layer, with a caveat that if the point falls onto or is close to a *P*_HMM,min_ plateau boundary, the larger of the two *P*_HMM,min_ values should be used.

Additionally, Figure 6 can be utilized to identify practical design constraints such as the total thickness of the material needed and the time required to fabricate the material. Using *P*_HMM,min_, *h*_ML,Ag–Ge_, and *h*_DL,Al_2_O_3__, the minimum total thickness for HMM fabrication, denoted as *h*_HMM,min_ is calculated using eq 7.

$$h_{\mathrm{HMM},\min}=P_{\mathrm{HMM},\min}(h_{\mathrm{ML},Ag-\mathrm{Ge}}+h_{\mathrm{DL},{\mathrm{Al}}_{2}O_{3}})+h_{\mathrm{PL},Al_{2}O_{3}}$$ 7

Three material combinations that exhibit λ_HMM_ ≈ 450 nm are chosen, indicated by the blue markers. The material combinations for the three points are summarized in Table 3, together with their total thickness (*h*_HMM,min_) and the approximated fabrication time (∼*t*_fab_). *t*_fab_ is calculated assuming an average growth rate of 0.3 Å/*s* and 10 min per material change in the evaporator. Typically, the choice of HMM fabrication is made based on application, i.e., choosing the wavelength and the type of HMM. Table 3 shows that the design chart can be used to identify several combinations of *h*_DL,Al_2_O_3__ and *h*_ML,Ag–Ge_ that lead to HMMs with λ_HMM_ ≈ 450 nm but have different total film thicknesses, number of periods, and fabrication times. Therefore, the combinations chosen for fabrication can now be determined by the overall thickness needed for the application or limited by the fabrication time available for production.

**Table 3:** Material Thickness Combinations for Blue Open Markers in Figure 6 with λ_HMM_ ≈ 450 nm and the Corresponding Period Number (*P*_HMM,min_), Total Film Thickness (*h*_HMM,min_), and Approximate Time of Fabrication (∼*t*_fab_)

marker	*h*_DL,Al_2_O_3__ (nm)	*h*_ML,Ag–Ge_ (nm)	*P*_HMM,min_	*h*_HMM,min_ (nm)	∼*t*_fab_ (h)
□	34.84	12.22	3	150	3.06
△	17.43	6.1	6	150	4.56
○	3.79	10.8	9	140.13	5.96

## Conclusions

Spectroscopic ellipsometry in conjunction with numerical simulations has been used to identify the onset of HMM character in layered Ag–Ge/Al_2_O_3_ films as a function of the number of periods, *P*_HMM,min_, using the convergence of the real part of the ratio of s- and p-polarized reflectivities obtained from **1P**, **4P**, and **7P** films. Subsequently, numerical simulations were used to apply the analysis to HMMs made from Ag–Ge and Al_2_O_3_ layers in the ranges of 1–20 and 1–40 nm, respectively. The emerging complex HMM design landscape is summarized as a chart that enables identification of the number of periods, layer/total thickness, and operational wavelength regime based on application, material, fabrication time, and cost. For instance, in super-resolution imaging applications, where ultrathin layers are usually required to reduce energy losses, one can refer to the bottom left corner of the design chart (see SI Section V, Figure S12).^59–61^ The middle of the design chart is useful in applications controlling spontaneous emission utilizing nanocavities, where thicker layers are permissible.^62,63^ Colloidal metamaterials that rely on core–shell nanoparticles may be more easily fabricated based on the upper third of the chart that requires low *P*_HMM,min_.^64^ More generally, analogous design charts can be created for other metal-dielectric combinations, such as gold/alumina^31^ and silver/titania,^65^ and are particularly impactful, as there is an ongoing search for alternative plasmonic materials that can be more easily synthesized and characterized.^66^

## Supplementary Material



## Notes

Additional data for ellipsometry calibration and optical constant measurements, details on the numerical simulation, comparison of ellipsometric response from **1P**, **4P**, and **7P** samples, and data for the validation of the numerical simulation, and zoomed-in/additional versions of the HMM design chart (PDF)
