# Supplementary material for: Ellipsometric Identification of Transition from a Layered Metal-Dielectric Film to a Hyperbolic Metamaterial
Source: ACS Appl Opt Mater. 2026 Jan 12;4(1):217–26. doi: 10.1021/acsaom.5c00534 (PMC12836358; doi:10.1021/acsaom.5c00534)
Supplement: Supplementary file 1 [file ot5c00534_si_001.pdf]

Supporting Information: Ellipsometric Identification of Transition from Layered Metal-dielectric Film to Hyperbolic Metamaterial

Samhita Kattekola,[a] Vinod M. Menon,[b] Alexander Couzis,[a] and Ilona Kretzschmar*[a]

[a]*Department of Chemical Engineering, City College of New York (CCNY), City University of New York (CUNY), 140th Street & Convent Avenue, New York, New York 10031, United States*
[b]*Department of Physics, City College of New York (CCNY), City University of New York (CUNY), 140th Street & Convent Avenue, New York, New York 10031, United States*

**E-mail: kretzschmar@ccny.cuny.edu*

The supporting information document provides additional data for calibration and optical constant measurements (Sec. I), details on the numerical simulation (Sec. II), comparison of ellipsometric response from **1P**, **4P**, and **7P** samples (Sec. III), data for the validation of the numerical simulation (Sec. IV) and versions of the design chart (Sec. V).

I Optical Constants Measurements and Thickness Calibration

Spectroscopic ellipsometry (SE) and atomic force microscopy (AFM) are used to obtain optical constants and calibrate layer thicknesses in this work.

1 AFM Measurement Procedure

AFM Measurements are made on selected samples to verify the correlation between nominal thickness (as reported by the quartz crystal monitor of the e-beam evaporator) and thickness obtained from fitting ellipsometry data of the deposited films.

1.1 AFM Sample Preparation

100 mm diameter single-side polished (SSP) silicon wafers purchased from University Wafer, Inc. are used as substrate. Every silicon wafer is cleaved into several pieces of approximately 1x1 cm^2, which then are cleaned by submerging them in a mixture of Nochromix® and sulfuric acid for two hours, followed by copious rinsing with deionized water and drying in an oven at 70°C. 4 μm silica particles are deposited onto the wafer surface. Silicon wafer pieces with 4 μm silica particle submonolayers are placed along with plain silicon wafers into the e-beam evaporator. After depositing the desired material using e-beam evaporation, the silica particles are brushed off. The silica particles shadow the substrate from the depositing material and leave empty circular areas on the substrate. The boundary between the shadowed area and the deposited film allows for accurate measurement of the film thickness (see Figure S1).

1.2 AFM Analysis

AFM analysis is performed using Gwyddion.[1] The obtained AFM data is corrected using Gwyddion's built-in Step-Line Correction function. Specific regions of interests (ROI) are then identified and the average step-height change is reported as thickness from at least 5 points on each sample.[1] An example of AFM data before and after processing is shown in Figures S1A and B, respectively.

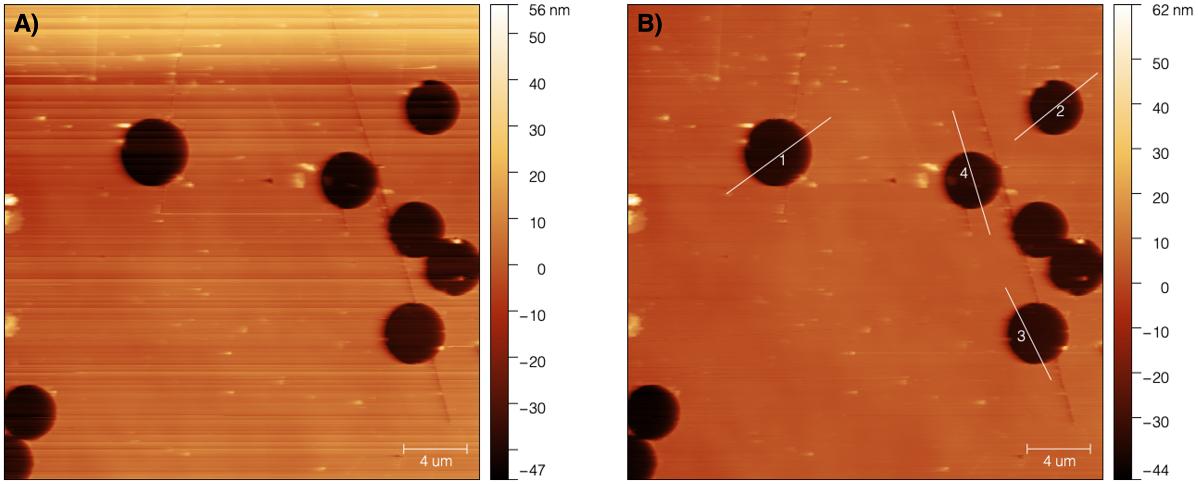

Figure S1: AFM data of 15 nm alumina film sample. (a) Raw AFM data obtained using Bruker Nanoscope. (b) Gwyddion processed AFM Data: Step-line correction applied, ROIs identified.

2 Spectroscopic Ellipsometry

This section provides additional details on general SE principles, systematic evaluation of optical parameters, and thickness calibrations. Optical constants obtained from ellipsometry are reported in this work as either ε or n and κ, which are related by Eqn. S1. Using n and κ values gives a feel for the transparency of a material, while ε values enable calculations of effective permittivities.

$$\varepsilon = \varepsilon_1 + i\varepsilon_2 \qquad \text{(Eqn. S1a)}$$

$$\varepsilon_1 = n^2 - \kappa^2 \qquad \text{(Eqn. S1b)}$$

$$\varepsilon_2 = 2n\kappa \qquad \text{(Eqn. S1c)}$$

For every sample, data is collected at a rate of 5 revs/measurement, which is the number of analyzer cycles per measurement. This rate is chosen as the smallest number to get reproducible data in the shortest amount of time.

2.1 Root mean square error (MSE)

J.A. Woollam Co., Inc. CompleteEASE® Software is used for modeling of optical constants. The regression analysis in CompleteEASE® Software computes the root mean square error (MSE) according to Eqn. S2, which can be used to identify the goodness-of-fit for the model.

$$MSE = \sqrt{\frac{1}{3w-m} \sum_{i=1}^{w} \left(\frac{N_{E_i} - N_{G_i}}{0.001}\right)^2 + \left(\frac{C_{E_i} - C_{G_i}}{0.001}\right)^2 + \left(\frac{S_{E_i} - S_{G_i}}{0.001}\right)^2} \quad \text{(Eqn. S2)}$$

where w is the number of wavelengths analyzed, m is the number of fit parameters, E and G subscripts indicate measured and model-generated data, respectively, and N, C, and S are given as:

$$N = \cos 2\Psi \quad \text{(Eqn. S3a)}$$

$$C = \sin 2\Psi \cos \Delta \quad \text{(Eqn. S3b)}$$

$$S = \sin 2\Psi \sin \Delta \quad \text{(Eqn. S3c)}$$

2.2 Silicon Wafer Modeling

Isotropic ellipsometry measurements on two pieces of silicon wafers are done over a wavelength range of 300-2000 nm with a 10 nm step size at incident angles of 65° and 75° using a Woollam V-Vase EllipsometerWVase® Software.[2] Data is analyzed and modeled with the native oxide on silicon wafer built-in model using the Woollam CompleteEase® Software.[3] The data reported is the average of two measurements with one standard deviation. The measured Ψ and Δ data for the silicon wafer substrate are shown as blue and red markers in Figure S2. The filled and open markers are measurements at 65° and 75° incident angle, respectively. The fit of the native oxide on silicon wafer is shown as a black solid line. MSE and average thickness of the native oxide on silicon wafer are reported in Table S1. The obtained parameters for native oxide thickness are locked and not allowed to vary during the modeling of other samples.

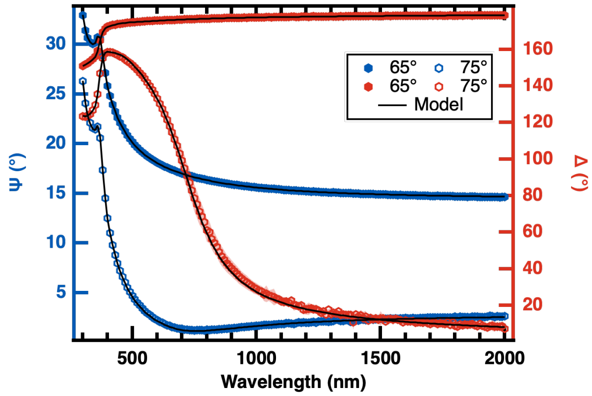

Figure S2: Ψ (blue markers, left axis) and Δ (red markers, right axis) for silicon wafer with native oxide are shown. Filled and open markers indicate 65° and 75° incident angles, respectively. Error bars are indicated by shaded areas and are generally smaller than the marker size. The black solid line shows the model for silicon wafer with native oxide.

Table S1: Ellipsometry characterization of cleaned silicon wafer substrate using CompleteEASE® silicon wafer with native oxide model

MSE	Native oxide thickness
3.0	2.1 ± 0.0 nm

2.3 Alumina (Al_2O_3) Films

The optical constants for alumina films are calibrated in two stages. Firstly, 15 nm Al_2O_3 films, are grown on four 1×1 cm^2 silicon wafers carrying 4 μm silica particles. The four pieces are distributed across the e-beam sample stage to account for any thickness variability across the sample stage. Utilizing the thickness obtained from the AFM measurements, literature values [4, 5] and ellipsometry fitting of the four samples, an optical model for alumina is developed by modifying the Cauchy Al_2O_3 built-in model in Woollam CompleteEase® Software.[3] The thickness measured by AFM and predicted by ellipsometry for the four 15 nm Al_2O_3 films are shown in Table S2.

Table S2: Ellipsometry characterization of Al_2O_3 films. $h_{Al_2O_3}$ measured by ellipsometry and AFM averaged over four Al_2O_3 films of 15 nm nominal thickness.

MSE	Nominal Thickness	$h_{Al_2O_3}$	$h_{total,AFM}$
5.0	15 nm	21.5 ± 0.4 nm	18 ± 2 nm

Subsequently, two sets of films with nominal thicknesses of 3, 6, 10, 15 and 20 nm of alumina are grown on silicon wafers. The calibration curve resulting from averaging of ellipsometry data for the 15 nm films (Table S2) and the two sets of calibration films are shown in Figure S3 as a function of nominal thickness. The AFM average from Table S2 is added as red marker.

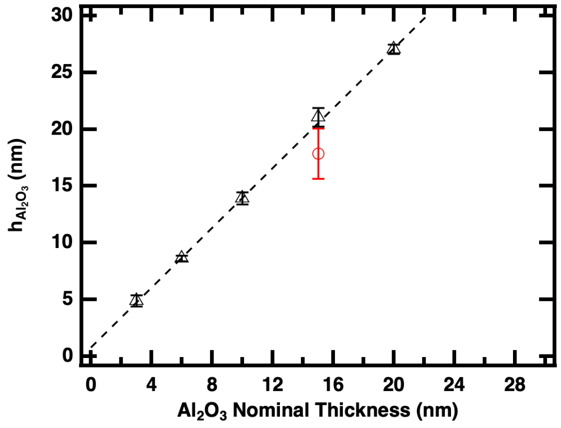

Figure S3: Al_2O_3 Film Calibration. $h_{Al_2O_3}$ from ellipsometry fitting of all Al_2O_3 films with nominal thicknesses of 3, 6, 10, 15 and 20 nm. The red open circle marker represents the average of the AFM thickness measurement from the four 15 nm film samples in Table S2.

The optical constants obtained from the ellipsometry data for Al_2O_3 films are shown in Figure S4.

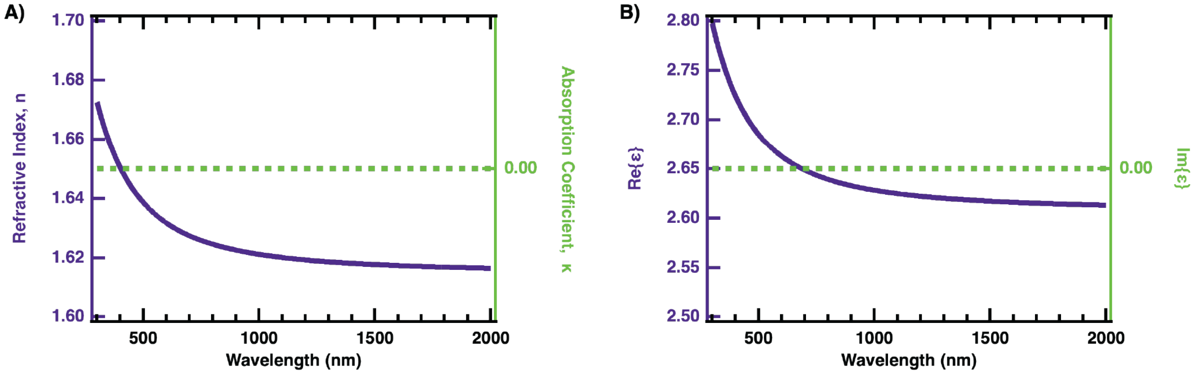

Figure S4: Al_2O_3 optical constants, A) refractive index, n, (solid line, violet left axis) and absorption coeffient, κ, (dashed line, green right axis) and B) real (solid line, violet left axis) and imaginary (dashed line, green right axis) part of ε.

2.4 Silver/Germanium (Ag−Ge) Films

Silver films of four nominal thicknesses (5, 10, 15, and 20 nm) are grown on a germanium seed layer (nominal thickness of 2 nm) and capped with a protection layer of Al_2O_3 (6 nm nominal thickness). The fitting for the Ag−Ge film is done in a manner similar to the work of Ciesielski et al.[6]. The silver layer used in the system is defined as a composite Ag−Ge layer and modeled using the CompleteEASE® general oscillator model. Drude and Lorentz oscillators are used along with one Cody-Lorentz oscillator. Drude and Lorentz oscillators capture the behavior of silver, while Cody-Lorentz oscillator captures that of germanium.

Table S3 shows the MSE, thickness measurements of Ag−Ge layer alone, h_{Ag-Ge}, and Ag−Ge/Al_2O_3 film from ellipsometry, and AFM measurements of the Ag−Ge/Al_2O_3 films. Figure S5 shows the calibration curve for the Ag−Ge/Al_2O_3 film correlating $h_{\mathrm{Ag-Ge/Al_2O_3}}$ with the nominal film thickness. In addition, the right axis (blue) shows the thickness of the Ag−Ge layer in the film. Red markers indicate AFM measurements of the total film.

The oscillator parameters are optimized on the silver calibration film with 10 nm nominal thickness, and held constant for all the other films, despite usually requiring adjustment.[7] Therefore, as the silver thickness increases, the MSE value increases (Table S3) leading to a slight overprediction of the silver thickness (Figure S5).

Table S3: Ellipsometry characterization of Ag−Ge films. $h_{\mathrm{Ag-Ge}}$ measured by ellipsometry and comparison of total silver calibration film thickness obtained from ellipsometry and AFM ($h_{\mathrm{Ag-Ge/Al_2O_3}}$). All thicknesses are given in nm.

MSE	Ag−Ge Nominal Thickness	h_{Ag-Ge}	Total Nominal Thickness	$h_{\mathrm{Ag-Ge/Al_2O_3}}$	$h_{total,AFM}$
2.3	12	10.4 ± 0.1	18	19.1 ± 0.2	19 ± 2
9.1	17	16.3 ± 0.1	23	25.0 ± 0.2	25 ± 1
13.0	22	23.7 ± 0.2	28	32.3 ± 0.3	28 ± 1

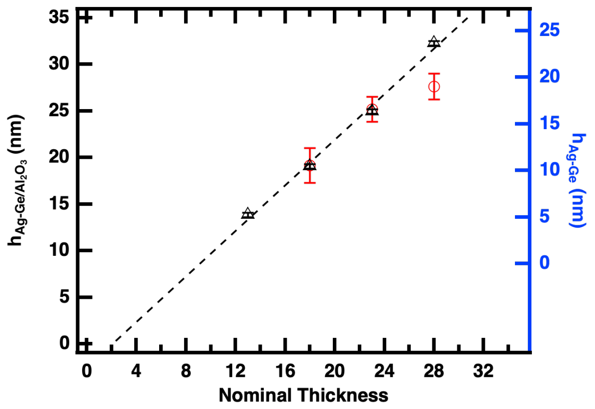

Figure S5: Ag−Ge Film Calibration. h_{Ag-Ge/Al_2O_3} from ellipsometry fitting for silver calibration films with nominal silver thicknesses of 5, 10, 15, and 20 nm. Blue axis (right) shows the corresponding silver layer thickness. The red open circle markers represent the AFM thickness measurements from Table S3.

The optical constant obtained from the ellipsometry data for Ag−Ge films are shown in Figure S6.

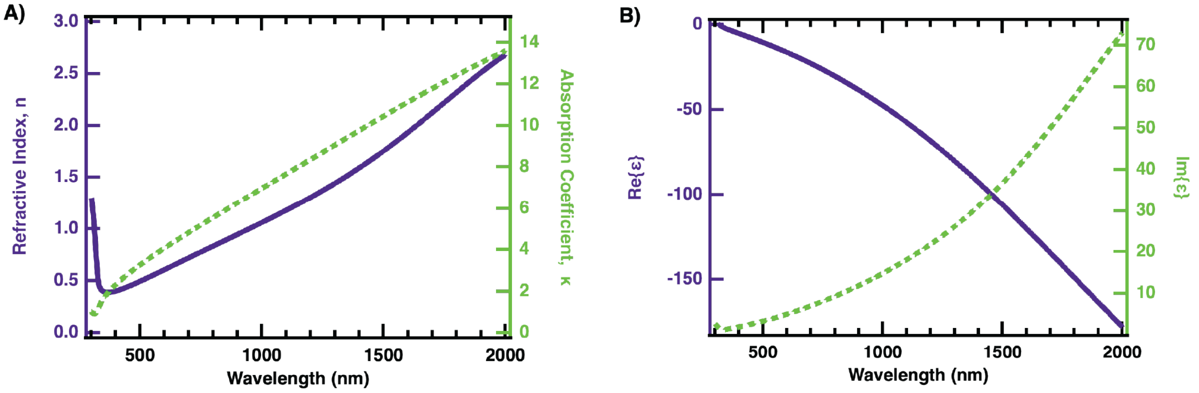

Figure S6: Ag−Ge optical constants, A) refractive index, *n*, (solid line, violet left axis) and absorption coeffient, *k*, (dashed line, green right axis) and B)real (solid line, green left axis) and imaginary (dashed line, violet right axis) part of ε.

3 Effective Medium Theory

The components of the effective permittivity for a system with Ag−Ge thickness of 10.84 nm and Al_2O_3 thickness of 21.1 nm are shown in Figure S7 with a transition wavelength of $\lambda_{HMM} \approx 403$ nm.

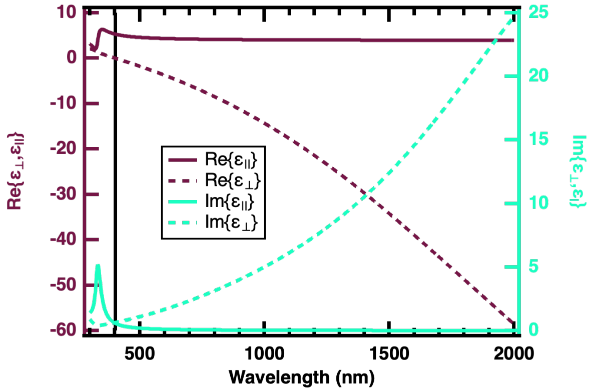

Figure S7: Real part (left axis, brown) and imaginary part (right axis, green) of $\varepsilon_{\|}$ (solid) and $\varepsilon_{\perp}$ (dashed) as a function of wavelength for 10.84 nm Ag−Ge and 21.1 nm Al_2O_3, respectively, predicted by EMT.

II Simulations: Abeles Transfer Matrix Method (TMM)

This section lists all the variables and formulas used to numerically simulate ellipsometry parameter, ρ using MATLAB®. The Transfer Matrix Method (TMM) used here is Abeles' formulation as outlined in [8]. Abeles' TMM is used as it sufficiently captures the propagation of light through the constituent layers, which are isotropic materials.

1 Variable List

$\mathbb{Z}$	set of integers
X_a	property X of material a
X_m	property X of metal, m
X_d	property X of dielectric, d
X_{amb}	property X of ambient material (air)
X_{subs}	property X of substrate material (silicon wafer)
$\tilde{X}$	complex variable, X
$\lvert\tilde{X}\rvert$ or $\lvert X\rvert$	magnitude of complex variable $\tilde{X}$ or absolute value of X
$arg(\tilde{X})$	phase of complex variable $\tilde{X}$
$Re(\tilde{X})$	real part of complex variable $\tilde{X}$
$Im(\tilde{X})$	imaginary part of complex variable $\tilde{X}$
$\tilde{X}^*$	complex conjugate of complex variable $\tilde{X}$
$\vec{X}$	vector variable, X
$\mathbf{X}$	tensor variable, X
ε	electric permittivity
μ	magnetic permeability
n	refractive index
κ	extinction coefficient
θ_i	incident angle
θ_t	transmission angle

k	wavenumber
ω	angular wave frequency
c	speed of light
λ	wavelength (nm)
λ_{HMM}	wavelength (nm) for onset of Type II hyperbolic dispersion in layered structure (nm)
h	thickness of layer (nm)
$h_{HMM,min}$	minimum total thickness of HMM (nm)
X_s	s-polarized ($\theta_u = 90°$) variable, X
X_p	p-polarized ($\theta_u = 0°$) variable, X
L	number of layers
P	number of periods
$P_{HMM,min}$	minimum number of periods for transition of layered structure into HMM
C_a	characteristic matrix of material a
M	total matrix for transfer matrix method
$\mathfrak{R}$	Fresnel reflection coefficient
$\mathfrak{T}$	fFesnel transmission coefficient
ρ	Ellipsometry parameter
ρ_∞	Ellipsometry parameter averaged over 100-150 periods

2 Refractive Index and Permittivity

$$\tilde{n}_a{}^2(\lambda) = \tilde{\varepsilon}_a(\lambda)\,\mu_a(\lambda) \tag{Eqn. S4}$$

For all materials in this work $\mu_a(\lambda) = 1$

$$\tilde{n}_a(\lambda) = n_a(\lambda) + i\kappa_a(\lambda) \tag{Eqn. S5}$$

$$\tilde{\varepsilon}_a(\lambda) = \varepsilon_{a_1}(\lambda) + i\varepsilon_{a_2}(\lambda) \tag{Eqn. S6}$$

$$\varepsilon_{a_1}(\lambda) = n_a^2(\lambda) - \kappa_a^2(\lambda) \tag{Eqn. S6a}$$

$$\varepsilon_{a_2}(\lambda) = 2n_a(\lambda)\,\kappa_a(\lambda) \tag{Eqn. S6b}$$

3 Transition Wavelength Calculation

$$\boldsymbol{\varepsilon}_{\mathbf{HMM}}(\lambda) = \begin{bmatrix} \tilde{\varepsilon}_{HMM_\parallel}(\lambda) & 0 & 0 \\ 0 & \tilde{\varepsilon}_{HMM_\parallel}(\lambda) & 0 \\ 0 & 0 & \tilde{\varepsilon}_{HMM_\perp}(\lambda) \end{bmatrix} \tag{Eqn. S7}$$

$$\Phi = \frac{h_m}{h_m + h_d} \tag{Eqn. S7a}$$

$$\tilde{\varepsilon}_{HMM_\perp}(\lambda) = \frac{\tilde{\varepsilon}_m \tilde{\varepsilon}_d}{\Phi\tilde{\varepsilon}_d + (1-\Phi)\tilde{\varepsilon}_m} \tag{Eqn. S7b}$$

$$\tilde{\varepsilon}_{HMM_\parallel}(\lambda) = \Phi\tilde{\varepsilon}_m + (1-\Phi)\tilde{\varepsilon}_d \tag{Eqn. S7c}$$

$$\lambda_{HMM} \approx min\left\{\lambda \in \mathbb{Z} \mid Re(\tilde{\varepsilon}_\parallel(\lambda)) < 0 \wedge Re(\tilde{\varepsilon}_\perp(\lambda)) > 0\right\} \tag{Eqn. S7d}$$

4 Snell's Law

$$\tilde{n}_{IncidentMedium}(\lambda)\sin\theta_i(\lambda) = \tilde{n}_{TransmittedMedium}(\lambda)\sin\theta_t(\lambda) \qquad \text{(Eqn. S8)}$$

5 Building the Characteristic Matrix

$$k(\lambda) = \frac{2\pi}{\lambda} \qquad \text{(Eqn. S9)}$$

$$\beta_a(\lambda) = k\tilde{n}_x h_a \cos\theta_{t_a} \qquad \text{(Eqn. S10)}$$

5.1 s-polarized wave

$$C_{s_x}(\lambda) = \begin{bmatrix} \cos\beta_a & \frac{-i}{s_a}\sin\beta_a \\ -is_a\sin\beta_a & \cos_a\beta_a \end{bmatrix} \qquad \text{(Eqn. S11)}$$

$$s_a(\lambda) = \sqrt{\frac{\tilde{\varepsilon}_a}{\mu}}\cos\theta_{t_a} \qquad \text{(Eqn. S11a)}$$

5.2 p-polarized wave

$$C_{p_a}(\lambda) = \begin{bmatrix} \cos\beta_a & \frac{-i}{p_a}\sin\beta_a \\ -ip_a\sin\beta_a & \cos\beta_a \end{bmatrix} \qquad \text{(Eqn. S12)}$$

$$p_a(\lambda) = \sqrt{\frac{\mu}{\tilde{\varepsilon}_a}}\cos\theta_{t_a} \qquad \text{(Eqn. S12a)}$$

6 Transfer Matrix Method

$$M_s(\lambda) = \prod_{l=1}^{L} C_{s_{a_l}} = \begin{bmatrix} S_{11}(\lambda) & S_{12}(\lambda) \\ S_{21}(\lambda) & S_{22}(\lambda) \end{bmatrix} \qquad \text{(Eqn. S13a)}$$

$$M_p(\lambda) = \prod_{l=1}^{L} C_{p_{a_l}} = \begin{bmatrix} P_{11}(\lambda) & P_{12}(\lambda) \\ P_{21}(\lambda) & P_{22}(\lambda) \end{bmatrix} \qquad \text{(Eqn. S13b)}$$

7 Fresnel Coefficients

$$\mathfrak{R}_s(\lambda) = \frac{s_{amb}S_{11} + s_{amb}s_{subs}S_{12} - S_{21} - s_{subs}S_{22}}{s_{amb}S_{11} + s_{amb}s_{subs}S_{12} + S_{21} + s_{subs}S_{22}} \qquad \text{(Eqn. S14a)}$$

$$\mathfrak{R}_p(\lambda) = \frac{p_{amb}P_{11} + p_{amb}p_{subs}P_{12} - P_{21} - p_{subs}P_{22}}{p_{amb}P_{11} + p_{amb}p_{subs}P_{12} + P_{21} + p_{subs}P_{22}} \qquad \text{(Eqn. S14b)}$$

$$\mathfrak{T}_s(\lambda) = \frac{2s_{amb}}{s_{amb}S_{11} + s_{amb}s_{subs}S_{12} + S_{21} + s_{subs}S_{22}} \qquad \text{(Eqn. S15a)}$$

$$\mathfrak{T}_p(\lambda) = \frac{2p_{amb}}{p_{amb}P_{11} + p_{amb}p_{subs}P_{12} + P_{21} + p_{subs}P_{22}} \qquad \text{(Eqn. S15b)}$$

8 Ellipsometry Parameters

$$\rho(\lambda) = \frac{\Re_p}{\Re_s} \quad \text{(Eqn. S16)}$$

9 Minimum Number of Periods Calculation

$$\rho_\infty = \frac{\sum_{L=100}^{150} \rho}{50} \quad \text{(Eqn. S17a)}$$

$$P_{HMM,min} = \min\{P \mid \rho_P(\lambda_{HMM}) - \rho_\infty(\lambda_{HMM}) \leq 0.035, P \in \mathbb{Z}\} \quad \text{(Eqn. S17b)}$$

10 Minimum Total Thickness Calculation

$$h_{HMM,min} = P_{HMM,min}(h_m + h_d) + 8.82 \quad \text{(Eqn. S18)}$$

8.82 nm is the thickness of the Al_2O_3 protection layer used uniformly throughout the simulations.

III Ellipsometry Comparison of 1P, 4P, and 7P Ellipsometry Responses

The **1P- 4P** (dashed line) and **4P- 7P** (solid line) film data differences $\Delta\Psi$ (blue, left axis) and $\Delta\Delta$ (red, right axis) obtained from Figures 3 and 4A and Figures 4A and B are displayed in Figure S8. Inspection of Figure S8 reveals that there is a significant residual for both Ψ and Δ for the **1P** and **4P** film comparison throughout the entire wavelength range, while the ellipsometric responses for the **4P** and **7P** films have a negligible difference above the transmission wavelength, $\lambda_{HMM} \approx 403$ nm.

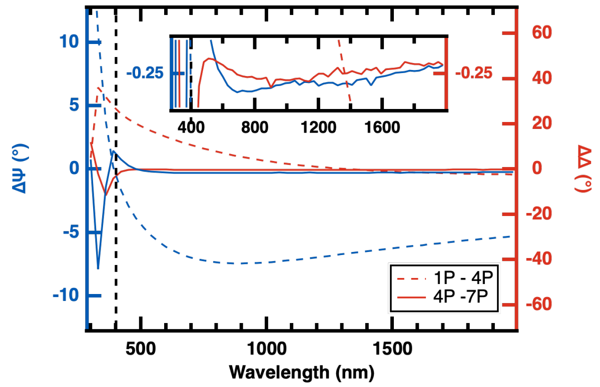

Figure S8: Difference $\Delta\Psi$ (blue axis) and $\Delta\Delta$ (red axis) for 65° obtained by subtracting **4P** from **1P** film (dashed lines) and **7P** from **4P** film (solid lines) ellipsometry average data shown in Figures 3 and 4A and 4A and 4B, respectively. Note that the range for both y-axes is equivalent to that of Figures 3 and 4 in the manuscript. The inset shows zoomed in region close to 0 for **4P - 7P** difference. Black dashed line indicates the transition wavelength of $\lambda_{HMM} \approx 403$ nm.

IV Numerical Simulation Model Validation

TMM as described in Sec.II is used to simulate the real ($\{\mathrm{Re}\{\rho\}\}$) and imaginary parts ($\mathrm{Im}\{\rho\}$) of the ellipsometric response for the **1P**, **4P**, and **7P** films using the optical constants for Ag−Ge and Al_2O_3 obtained form the calibrations described in Sec.I. The simulations overlaid with experimental data for **1P**, **4P**, and **7P** are shown in Figures S9, S10, and S11, respectively. The real and imaginary parts of ρ are shown as pink (left axis) and olive (right axis) markers, respectively. The filled and open markers indicate 65° and 75° incident angles, respectively. The solid vertical line indicates the transition wavelength, $\lambda_{HMM} \approx 403$ nm for the system. The wavelength range for both simulation and experimental data is 300 nm to 2000 nm. The step size for experimental measurements is 30 nm, while the step size for simulations is 1 nm. The experimental data for each type of film is averaged over three independently fabricated samples with one standard deviation (shown as shaded areas in the figures). The agreement between simulation and experimental data in Figures S9, S10, and S11 validates the numerical simulations.

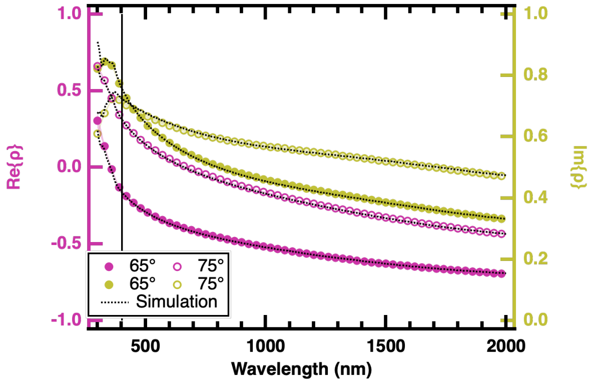

Figure S9: Comparison of $\mathrm{Re}\{\rho\}$ (pink, left axis) and $\mathrm{Im}\{\rho\}$ (olive, right axis) obtained from ellipsometry (markers) and numerical simulation with TMM (dotted line) for **1P** film sample. Solid line indicates the transition wavelength, $\lambda_{HMM} \approx 403$ nm to Type II HMM.

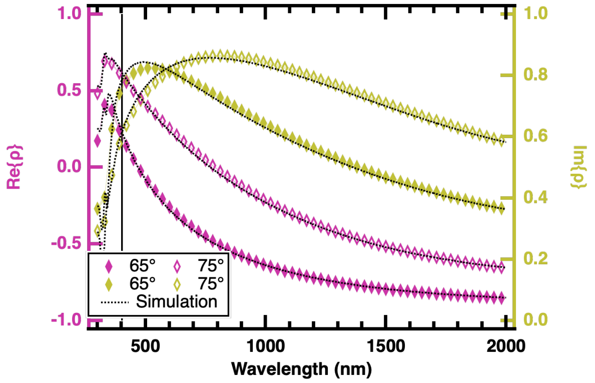

Figure S10: Comparison of $\mathrm{Re}\{\rho\}$ (pink, left axis) and $\mathrm{Im}\{\rho\}$ (olive, right axis) obtained ellipsometry (markers) and numerical simulation with TMM (dotted line) for **4P** film sample. Solid line indicates the transition wavelength, $\lambda_{HMM} \approx 403$ nm to Type II HMM.

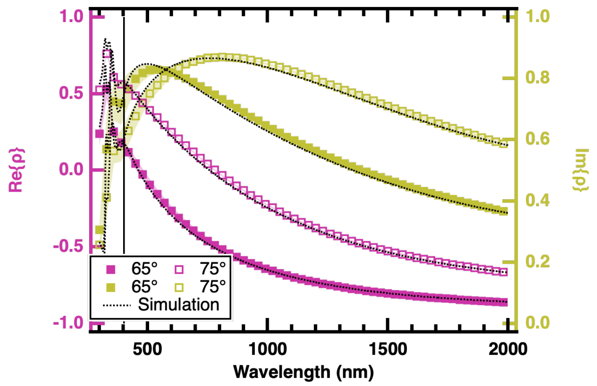

Figure S11: Comparison of Re$\{\rho\}$ (pink, left axis) and Im$\{\rho\}$ (olive, right axis) obtained from ellipsometry (markers) and numerical simulation with TMM (dotted line) for **7P** film sample. Solid line indicates the transition wavelength, $\lambda_{HMM} \approx 403$ nm to Type II HMM

V Design Chart

Zoomed-in versions at lower silver and dielectric layer thickness and additional variations of Figure 6 mentioned in the main text discussion are provided in Figures S12 - S15. All charts are based on the same optical constants and numerical approach discussed in the main text. Where appropriate, the thickness combination used in this work ($\Phi = 0.34$) and its transition wavelength ($\lambda \approx 403$ nm) are marked with a red star and dotted line, respectively.

1 Very thin metal layers (dielectric-rich region)

Figure S12 shows a zoomed-in version of the dielectric-rich region of Figure 6 in the main text. Colored lines indicate the λ_{HMM} at which the film displays hyperbolic dispersion. Grey solid lines (same as black solid lines in Figure 6) indicate the change in $P_{HMM,min}$. The last gray line is $P_{HMM,min} = 40$, which is the largest number of periods for this combination of metal-dielectric materials and occurs for $h_{\mathrm{Ag-Ge}} = 0.01$ to $h_{\mathrm{Ag-Ge}} = 0.03$ and $h_{\mathrm{Al_2O_3}} = 0.01$ to $h_{\mathrm{Al_2O_3}} = 0.12$. Layer thicknesses in these ranges have been reported for samples grown using atomic layer deposition (ALD).[9]

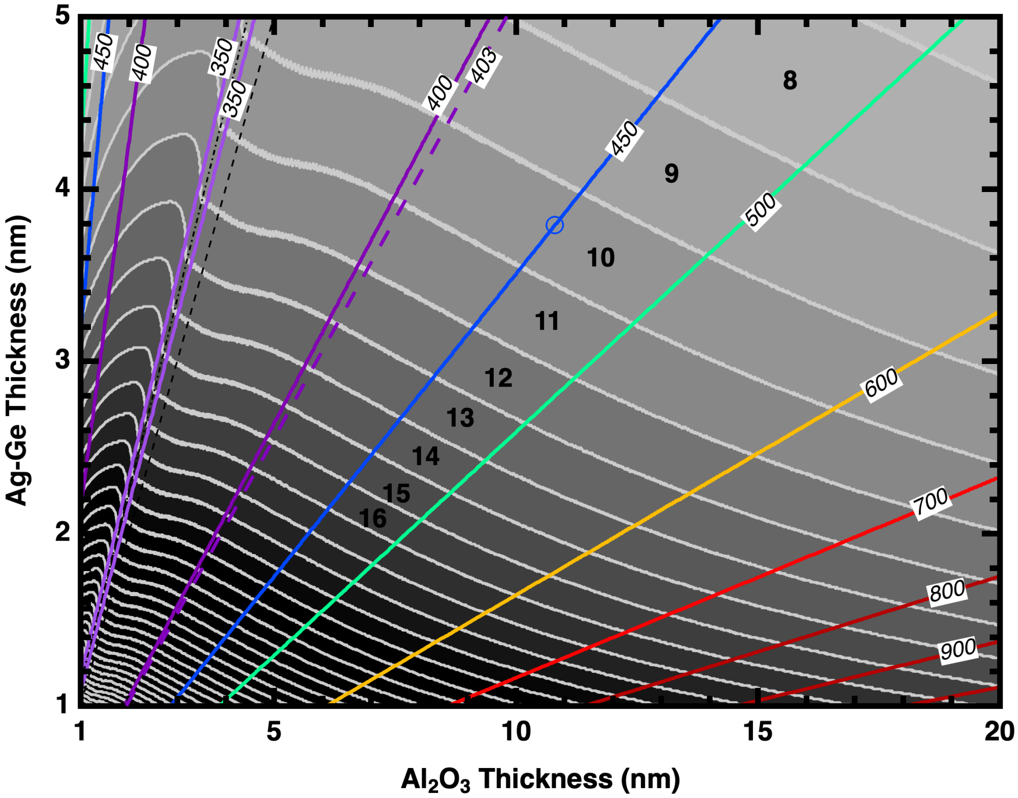

Figure S12: HMM Design Chart displaying the dielectric-rich region ($h_m = 1$ to 5 nm). Colored lines indicate selected λ_{HMM} at which the film displays hyperbolic dispersion, respectively. Gray lines indicate the change in $P_{HMM,min}$ by one.Blue open marker indicates one of the combinations shown in Table 3 (see main text).

2 Very thin dielectric layers (metal-rich region)

Figure S13 shows a zoomed-in version of the metal-rich region of Figure 6 in the main text, which shows additional features in $P_{HMM,min}$ contours discussed in the text such as the sliver where $P_{HMM,min} = 1$. It also reveals an additional pocket of $P_{HMM,min} = 3$ between $h_m = 6$ to 15 nm and $h_d = 1$ to 1.3 nm, which is the only region of the chart where HMM materials with the same Al_2O_3 layer thickness but differing Ag−Ge layer thickness can be found.

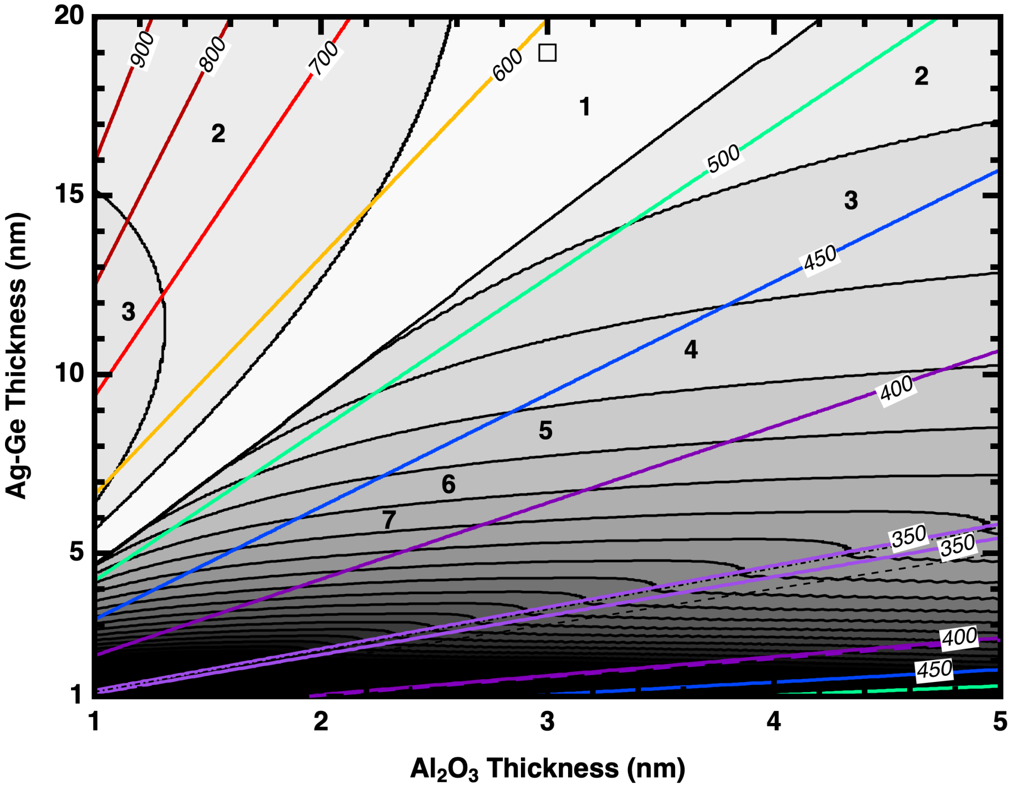

Figure S13: HMM Design Chart displaying the metal-rich region ($h_d = 1$ to 5 nm). Colored lines indicate selected λ_{HMM} at which the film displays hyperbolic dispersion, respectively. The black square marker is for $h_{\text{Ag-Ge}} = 19$ nm and $h_{\text{Al}_2\text{O}_3} = 4$ nm (see main text). Note that there is also a pocket with $P_{HMM,min} = 3$ at low $h_{\text{Al}_2\text{O}_3}$ (see text).

3 Number of periods and total thickness

Figure S14 shows the design chart as a function of $P_{HMM,min}$ and $h_{HMM,min}$. The blue open markers are the three combinations chosen with $\lambda_{HMM} \approx 450$ nm (see Table 3 and Figure 6 in main text). In this graph, total thickness contour lines coincide with the $P_{HMM,min}$ boundaries.

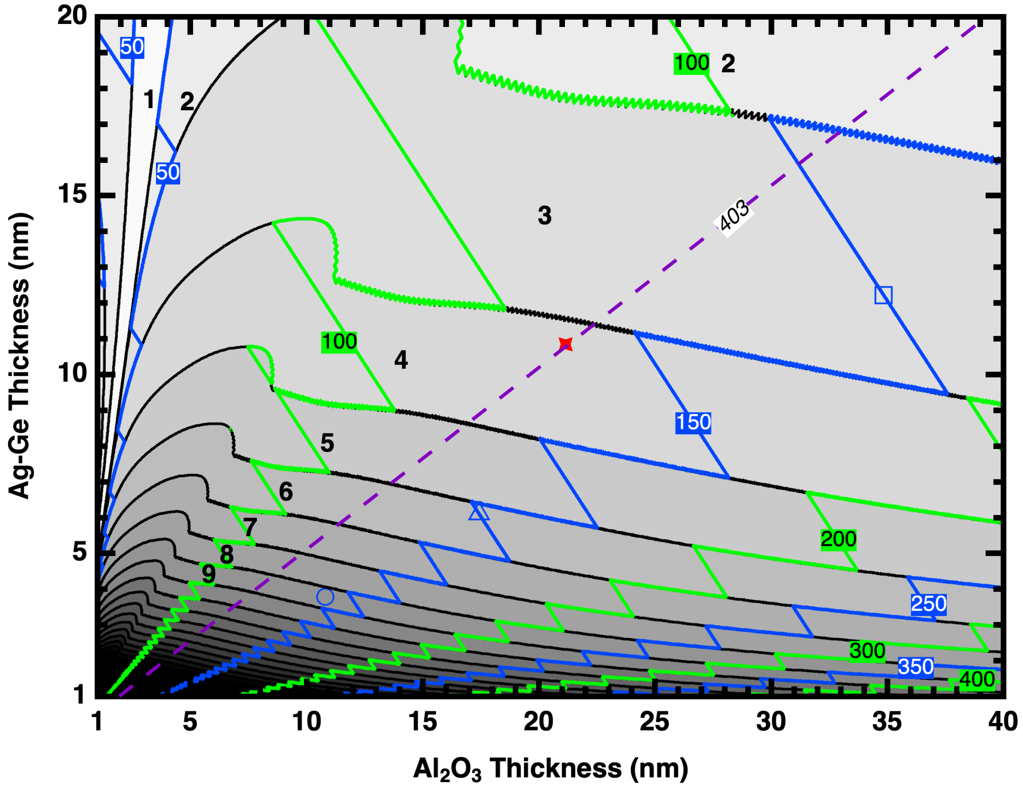

Figure S14: HMM Design Chart displaying $P_{HMM,min}$ and contours of constant thickness (colored lines). Note that the open blue triangle and rectangle fall on the same 150 nm thickness contour line (see main text).

4 Number of periods and fill fraction

Figure S15 shows the design chart as a function of $P_{HMM,min}$ and Φ. The blue lines indicate contours of Φ. Black dot-dashed line represents $\Phi = 0.53$, which is the contour line for $\lambda_{HMM,min} \approx 348$ nm. Blue dashed line represents $\Phi = 0.5$ (see main text).

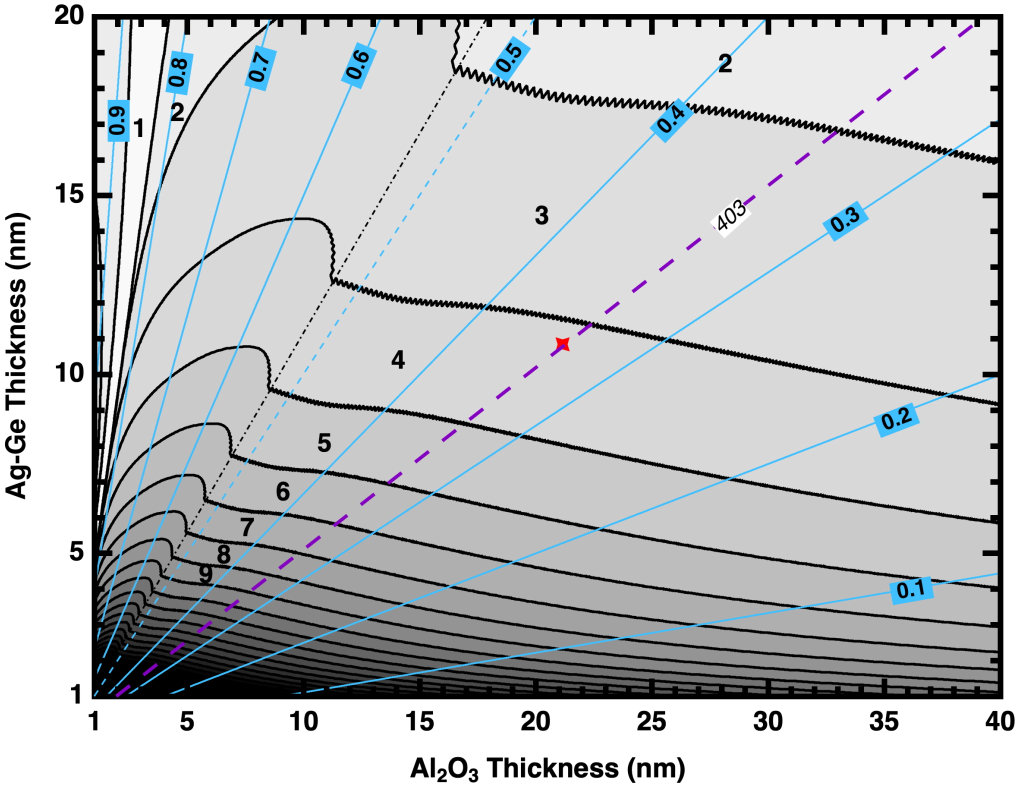

Figure S15: HMM Design Chart displaying $P_{HMM,min}$ and contours of metal fill fraction, Φ, as blue solid lines. Black dashed line indicates the fill fraction for $\lambda_{HMM,min} \approx 348$ nm (see main text).

References

[1] David Nečas and Petr Klapetek, "Gwyddion: an open-source software for SPM data analysis", *Central European Journal of Physics* **10**, pp. 181–188 (2012).

[2] J.A. Woollam Co., Inc., "Wvase®" (2020), J.A. Woollam Co., Inc., Lincoln, NE.

[3] J.A. Woollam Co., Inc., "CompleteEase®" (2020), J.A. Woollam Co., Inc., Lincoln, NE.

[4] J. Houska, J. Blazek, J. Rezek, and S. Proksova, "Overview of optical properties of Al2O3 films prepared by various techniques", *Thin Solid Films* **520**(16), pp. 5405–5408 (2012).

[5] T. S. Eriksson, A. Hjortsberg, G. A. Niklasson, and C. G. Granqvist, "Infrared optical properties of evaporated alumina films", *Applied Optics* **20**(15), pp. 2742 (1981).

[6] Arkadiusz Ciesielski, Lukasz Skowronski, Marek Trzcinski, and Tomasz Szoplik, "Controlling the optical parameters of self-assembled silver films with wetting layers and annealing", *Applied Surface Science* **421**, pp. 349–356 (2017).

[7] Guowen Ding, César Clavero, Daniel Schweigert, and Minh Le, "Thickness and microstructure effects in the optical and electrical properties of silver thin films", *AIP Advances* **5**(11), pp. 117234 (2015).

[8] Max Born and Emil Wolf, *Principles of Optics: Electromagnetic Theory of Propagation, Interference and Diffraction of Light*, Cambridge University Press 7 edition (1999).

[9] Priscilla Kelly, Mingzhao Liu, and Lyuba Kuznetsova, "Designing optical metamaterial with hyperbolic dispersion based on an al:zno/zno nano-layered structure using the atomic layer deposition technique", *Appl. Opt.* **55**(11), pp. 2993–2997 (2016).
